# *catena*-Poly[[tribenzyl­tin(IV)]-μ-(*E*)-3-(2-fur­yl)prop-2-enoato-κ^2^ *O*:*O*′]

**DOI:** 10.1107/S1600536811023919

**Published:** 2011-06-25

**Authors:** Thy Chun Keng, Kong Mun Lo, Seik Weng Ng

**Affiliations:** a Department of Chemistry, University of Malaya, 50603 Kuala Lumpur, Malaysia

## Abstract

In the title carboxyl­ate-bridged polymer, [Sn(C_7_H_7_)_3_(C_7_H_5_O_3_)]_*n*_, the Sn^IV^ atom exists in a distorted *trans*-C_3_SnO_2_ trigonal–bipyramidal geometry. The polymer propagates as a chain along the *a* axis. There are two independent formula units in the asymmetric unit; the furyl ring of one of the anions is disordered over two positions in a 0.630 (8):0.370 (8) ratio. The crystal studied was a non-merohedral twin with a minor twin domain of 37.3 (1)%.

## Related literature

For a related tribenzyl­tin cinnamate, see: Lo & Ng (2011 ▶).
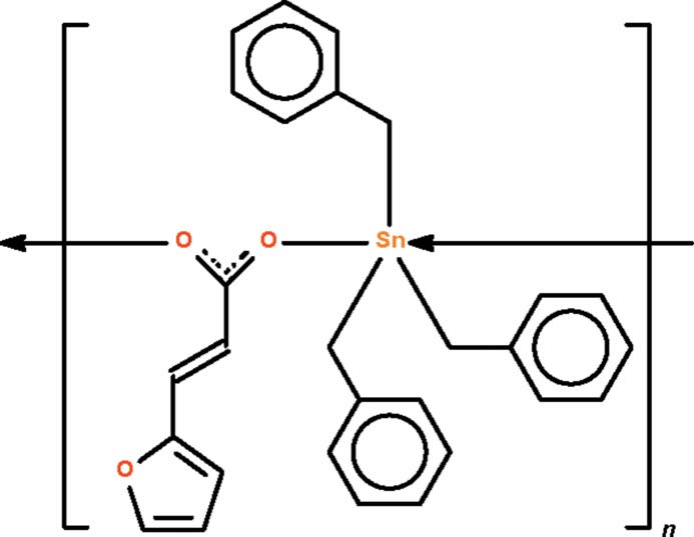


## Experimental

### 

#### Crystal data

[Sn(C_7_H_7_)_3_(C_7_H_5_O_3_)]
*M* *_r_* = 529.18
Triclinic, 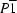

*a* = 10.5744 (1) Å
*b* = 10.8946 (1) Å
*c* = 21.4199 (3) Å
α = 101.7132 (6)°
β = 90.6861 (6)°
γ = 101.2031 (6)°
*V* = 2366.83 (5) Å^3^
*Z* = 4
Mo *K*α radiation
μ = 1.11 mm^−1^
*T* = 100 K
0.30 × 0.20 × 0.10 mm

#### Data collection

Bruker SMART APEX diffractometer
Absorption correction: multi-scan (*TWINABS*; Bruker, 2009 ▶) *T*_min_ = 0.574, *T*_max_ = 0.746
40884 measured reflections
11649 independent reflections
10010 reflections with *I* > 2σ(*I*)
*R*_int_ = 0.030

#### Refinement

*R*[*F* ^2^ > 2σ(*F* ^2^)] = 0.033
*wR*(*F* ^2^) = 0.106
*S* = 1.07
11649 reflections
603 parameters
56 restraints
H-atom parameters constrained
Δρ_max_ = 0.80 e Å^−3^
Δρ_min_ = −0.58 e Å^−3^

### 

Data collection: *APEX2* (Bruker, 2009 ▶); cell refinement: *SAINT* (Bruker, 2009 ▶); data reduction: *SAINT*; program(s) used to solve structure: *SHELXS97* (Sheldrick, 2008 ▶); program(s) used to refine structure: *SHELXL97* (Sheldrick, 2008 ▶); molecular graphics: *X-SEED* (Barbour, 2001 ▶); software used to prepare material for publication: *publCIF* (Westrip, 2010 ▶).

## Supplementary Material

Crystal structure: contains datablock(s) global, I. DOI: 10.1107/S1600536811023919/xu5233sup1.cif

Structure factors: contains datablock(s) I. DOI: 10.1107/S1600536811023919/xu5233Isup2.hkl

Additional supplementary materials:  crystallographic information; 3D view; checkCIF report

## Figures and Tables

**Fig. 1.**
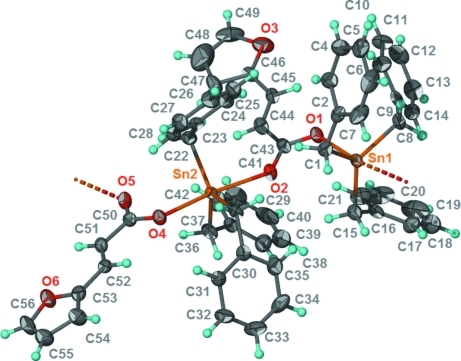
Thermal ellipsoid plot (Barbour, 2001) of a portion of the polymeric chain structure of [Sn(C7H7)3(C7H5O3)]n at the 70% probability level; hydrogen atoms are drawn as spheres of arbitrary radius. The disorder in one of the furyl rings is not shown.

## supplementary crystallographic information

### Comment

We have reported tribenzyltin 3-phenylprop-2-enoate recently; the compound exists as a carboxyate bridged polymer whose repeat distance is half the *b*-axial length (Lo & Ng, 2011). Replacing the phenyl unit by a furyl unit gives the corresponding polymeric compound (Scheme I). The Sn^IV^ atom in [Sn(C_7_H_7_)_3_(C_7_H_5_O_2_)]*_n_* exists in a *trans*-C_3_SnO_2_ trigonal-bipyramidal geometry (Fig. 1). The polymer propagates as a chain along the *a*-axis with a repeat distance that is half the *a*-axial length.

### Experimental

Tribenzyltin hydroxide (0.4 g, 1 mmol) and furylacrylic acid (0.14 g, 1 mmol) were heated in ethanol (50 ml) for 2 h. The solution was filtered; evaporation of the solvent gave colorless crystals.

### Refinement

Carbon-bound H-atoms were placed in calculated positions (C—H 0.95 to 0.98 Å) and were included in the refinement in the riding model approximation, with *U*(H) set to 1.2*U*(C).

One of the furan rings is disordered over two positions. The furan ring was refined subject to the atoms being restrained to a bonding distance of 1.35±0.01 Å. The ring was restrained to near planarity. Because the ring is disordered so that the O atom overlapped with a C atom, the temperature factors of O3 were set to those of C47. Those of O3' were set to those of C47'. Additionally the temperature factors of the disordered atoms were restrained to be nearly isotropic. In view of the twinned nature of the crystal, this treatment was considered adequate.

### Crystal data

**Table:**

[Sn(C_7_H_7_)_3_(C_7_H_5_O_2_)]	*Z* = 4
*M**_r_* = 529.18	*F*(000) = 1072
Triclinic, *P*1	*D*_x_ = 1.485 Mg m^−^^3^
Hall symbol: -P 1	Mo *K*α radiation, λ = 0.71073 Å
*a* = 10.5744 (1) Å	Cell parameters from 4375 reflections
*b* = 10.8946 (1) Å	θ = 2.4–28.2°
*c* = 21.4199 (3) Å	µ = 1.11 mm^−^^1^
α = 101.7132 (6)°	*T* = 100 K
β = 90.6861 (6)°	Block, colorless
γ = 101.2031 (6)°	0.30 × 0.20 × 0.10 mm
*V* = 2366.83 (5) Å^3^	

### Data collection

**Table:**

Bruker SMART APEX diffractometer	11649 independent reflections
Radiation source: fine-focus sealed tube	10010 reflections with *I* > 2σ(*I*)
graphite	*R*_int_ = 0.030
ω scans	θ_max_ = 28.3°, θ_min_ = 1.0°
Absorption correction: multi-scan (TWINABS; Bruker, 2009)	*h* = −14→14
*T*_min_ = 0.574, *T*_max_ = 0.746	*k* = −14→14
40884 measured reflections	*l* = 0→28

### Refinement

**Table:**

Refinement on *F*^2^	Primary atom site location: structure-invariant direct methods
Least-squares matrix: full	Secondary atom site location: difference Fourier map
*R*[*F*^2^ > 2σ(*F*^2^)] = 0.033	Hydrogen site location: inferred from neighbouring sites
*wR*(*F*^2^) = 0.106	H-atom parameters constrained
*S* = 1.07	*w* = 1/[σ^2^(*F*_o_^2^) + (0.0595*P*)^2^ + 1.754*P*] where *P* = (*F*_o_^2^ + 2*F*_c_^2^)/3
11649 reflections	(Δ/σ)_max_ = 0.001
603 parameters	Δρ_max_ = 0.80 e Å^−^^3^
56 restraints	Δρ_min_ = −0.58 e Å^−^^3^

### Fractional atomic coordinates and isotropic or equivalent isotropic displacement parameters (Å^2^)

**Table:**

	*x*	*y*	*z*	*U*_iso_*/*U*_eq_	Occ. (<1)
Sn1	0.42162 (2)	0.46489 (2)	0.738222 (11)	0.01823 (7)	
Sn2	0.90414 (2)	0.38862 (2)	0.779954 (10)	0.01697 (6)	
O1	0.5896 (2)	0.5262 (2)	0.68649 (12)	0.0240 (5)	
O2	0.7317 (2)	0.4722 (2)	0.74811 (12)	0.0237 (5)	
O3	0.8566 (8)	0.6995 (6)	0.5163 (3)	0.0385 (11)	0.630 (8)
O3'	1.0107 (8)	0.6534 (10)	0.5614 (5)	0.0320 (15)	0.370 (8)
O4	1.0461 (2)	0.2949 (2)	0.81974 (11)	0.0219 (5)	
O5	1.2189 (2)	0.4037 (2)	0.78297 (13)	0.0252 (5)	
O6	1.4103 (3)	0.2200 (3)	0.95100 (14)	0.0347 (6)	
C1	0.4418 (3)	0.2674 (3)	0.72014 (17)	0.0227 (7)	
H1A	0.5333	0.2630	0.7130	0.027*	
H1B	0.4168	0.2309	0.7580	0.027*	
C2	0.3595 (4)	0.1903 (3)	0.66322 (17)	0.0237 (7)	
C3	0.3951 (4)	0.2041 (4)	0.60194 (18)	0.0292 (8)	
H3	0.4709	0.2637	0.5971	0.035*	
C4	0.3213 (4)	0.1319 (4)	0.5482 (2)	0.0361 (9)	
H4	0.3474	0.1421	0.5070	0.043*	
C5	0.2106 (4)	0.0457 (4)	0.5541 (2)	0.0407 (11)	
H5	0.1607	−0.0044	0.5172	0.049*	
C6	0.1734 (4)	0.0332 (4)	0.6138 (2)	0.0456 (12)	
H6	0.0964	−0.0253	0.6182	0.055*	
C7	0.2461 (4)	0.1045 (4)	0.6682 (2)	0.0374 (10)	
H7	0.2180	0.0946	0.7092	0.045*	
C8	0.2893 (3)	0.5092 (4)	0.67325 (18)	0.0238 (7)	
H8A	0.2456	0.4273	0.6455	0.029*	
H8B	0.2222	0.5441	0.6990	0.029*	
C9	0.3369 (3)	0.6001 (3)	0.63018 (18)	0.0224 (7)	
C10	0.3693 (4)	0.5560 (4)	0.56875 (19)	0.0322 (8)	
H10	0.3683	0.4673	0.5545	0.039*	
C11	0.4031 (4)	0.6374 (4)	0.52738 (19)	0.0352 (9)	
H11	0.4257	0.6048	0.4853	0.042*	
C12	0.4040 (4)	0.7672 (4)	0.5474 (2)	0.0359 (9)	
H12	0.4240	0.8236	0.5188	0.043*	
C13	0.3752 (4)	0.8130 (4)	0.6098 (2)	0.0337 (9)	
H13	0.3776	0.9018	0.6244	0.040*	
C14	0.3431 (3)	0.7301 (4)	0.65082 (18)	0.0266 (7)	
H14	0.3251	0.7628	0.6936	0.032*	
C15	0.4923 (4)	0.6006 (3)	0.82487 (18)	0.0253 (7)	
H15A	0.4448	0.5740	0.8610	0.030*	
H15B	0.5848	0.6010	0.8330	0.030*	
C16	0.4768 (3)	0.7330 (3)	0.82155 (17)	0.0235 (7)	
C17	0.3733 (4)	0.7818 (4)	0.84802 (19)	0.0296 (8)	
H17	0.3104	0.7301	0.8681	0.036*	
C18	0.3602 (5)	0.9046 (4)	0.8456 (2)	0.0430 (11)	
H18	0.2892	0.9370	0.8645	0.052*	
C19	0.4491 (6)	0.9797 (4)	0.8162 (2)	0.0505 (13)	
H19	0.4404	1.0644	0.8150	0.061*	
C20	0.5514 (5)	0.9321 (4)	0.7882 (2)	0.0463 (12)	
H20	0.6125	0.9834	0.7671	0.056*	
C21	0.5648 (4)	0.8086 (4)	0.7909 (2)	0.0352 (9)	
H21	0.6351	0.7759	0.7715	0.042*	
C22	0.9459 (3)	0.3131 (4)	0.68307 (17)	0.0238 (7)	
H22A	0.9291	0.3706	0.6550	0.029*	
H22B	1.0377	0.3059	0.6809	0.029*	
C23	0.8606 (3)	0.1845 (3)	0.66224 (15)	0.0217 (7)	
C24	0.7443 (3)	0.1658 (4)	0.62716 (17)	0.0258 (7)	
H24	0.7196	0.2364	0.6141	0.031*	
C25	0.6641 (4)	0.0466 (4)	0.61101 (18)	0.0308 (8)	
H25	0.5853	0.0361	0.5869	0.037*	
C26	0.6981 (4)	−0.0582 (4)	0.62976 (19)	0.0315 (8)	
H26	0.6429	−0.1402	0.6187	0.038*	
C27	0.8137 (4)	−0.0412 (4)	0.66493 (19)	0.0306 (8)	
H27	0.8386	−0.1122	0.6776	0.037*	
C28	0.8928 (4)	0.0788 (4)	0.68148 (18)	0.0278 (8)	
H28	0.9705	0.0896	0.7065	0.033*	
C29	0.7597 (3)	0.2648 (3)	0.82037 (16)	0.0225 (7)	
H29A	0.6739	0.2601	0.7999	0.027*	
H29B	0.7782	0.1774	0.8121	0.027*	
C30	0.7579 (3)	0.3144 (3)	0.89148 (17)	0.0223 (7)	
C31	0.8265 (4)	0.2693 (4)	0.93461 (17)	0.0257 (7)	
H31	0.8708	0.2020	0.9195	0.031*	
C32	0.8310 (4)	0.3213 (4)	0.99948 (18)	0.0341 (9)	
H32	0.8781	0.2895	1.0285	0.041*	
C33	0.7671 (5)	0.4195 (4)	1.02212 (19)	0.0390 (10)	
H33	0.7713	0.4557	1.0665	0.047*	
C34	0.6977 (5)	0.4647 (4)	0.9803 (2)	0.0379 (10)	
H34	0.6535	0.5319	0.9958	0.045*	
C35	0.6922 (4)	0.4123 (4)	0.91577 (19)	0.0303 (8)	
H35	0.6430	0.4433	0.8873	0.036*	
C36	0.9931 (4)	0.5649 (3)	0.84266 (18)	0.0262 (8)	
H36A	0.9627	0.5604	0.8858	0.031*	
H36B	1.0871	0.5669	0.8448	0.031*	
C37	0.9757 (3)	0.6910 (3)	0.82988 (16)	0.0212 (6)	
C38	0.8917 (4)	0.7590 (4)	0.8639 (2)	0.0316 (8)	
H38	0.8397	0.7227	0.8940	0.038*	
C39	0.8824 (5)	0.8794 (4)	0.8546 (2)	0.0393 (10)	
H39	0.8242	0.9245	0.8783	0.047*	
C40	0.9565 (5)	0.9337 (4)	0.8116 (2)	0.0404 (10)	
H40	0.9518	1.0173	0.8063	0.048*	
C41	1.0376 (4)	0.8667 (4)	0.7762 (2)	0.0416 (10)	
H41	1.0872	0.9028	0.7453	0.050*	
C42	1.0476 (4)	0.7459 (4)	0.7853 (2)	0.0329 (9)	
H42	1.1045	0.7005	0.7606	0.040*	
C43	0.7049 (3)	0.5203 (3)	0.70187 (17)	0.0211 (7)	
C44	0.8079 (3)	0.5711 (3)	0.66284 (18)	0.0255 (7)	
H44	0.8951	0.5766	0.6763	0.031*	
C45	0.7842 (3)	0.6097 (3)	0.60954 (17)	0.0225 (7)	
H45	0.6971	0.6101	0.5983	0.027*	
C46	0.8821 (3)	0.6510 (3)	0.56786 (16)	0.0246 (7)	
C47	1.0034 (7)	0.6364 (8)	0.5815 (4)	0.0320 (15)	0.630 (8)
H47	1.0361	0.6047	0.6152	0.038*	0.630 (8)
C48	1.0654 (10)	0.6819 (9)	0.5313 (5)	0.069 (3)	0.630 (8)
H48	1.1548	0.6866	0.5247	0.083*	0.630 (8)
C49	0.9835 (10)	0.7188 (9)	0.4930 (4)	0.047 (3)	0.630 (8)
H49	1.0068	0.7524	0.4563	0.057*	0.630 (8)
C47'	0.8439 (17)	0.6976 (12)	0.5179 (5)	0.0385 (11)	0.37
H47'	0.7578	0.7069	0.5105	0.046*	0.370 (8)
C48'	0.9424 (11)	0.7300 (13)	0.4793 (6)	0.020 (3)	0.370 (8)
H48'	0.9379	0.7641	0.4420	0.025*	0.370 (8)
C49'	1.0479 (11)	0.7022 (10)	0.5062 (5)	0.022 (3)	0.370 (8)
H49'	1.1322	0.7136	0.4907	0.027*	0.370 (8)
C50	1.1687 (3)	0.3309 (3)	0.81872 (17)	0.0214 (7)	
C51	1.2508 (3)	0.2870 (3)	0.86180 (17)	0.0225 (7)	
H51	1.3418	0.3130	0.8613	0.027*	
C52	1.2036 (4)	0.2129 (3)	0.90133 (16)	0.0240 (7)	
H52	1.1127	0.1834	0.8994	0.029*	
C53	1.2796 (4)	0.1736 (4)	0.94699 (18)	0.0281 (8)	
C54	1.2487 (5)	0.0981 (5)	0.9901 (2)	0.0388 (10)	
H54	1.1650	0.0546	0.9974	0.047*	
C55	1.3673 (5)	0.0976 (5)	1.0223 (2)	0.0429 (11)	
H55	1.3782	0.0537	1.0554	0.051*	
C56	1.4594 (5)	0.1702 (5)	0.9971 (2)	0.0439 (11)	
H56	1.5486	0.1855	1.0096	0.053*	

### Atomic displacement parameters (Å^2^)

**Table:**

	*U*^11^	*U*^22^	*U*^33^	*U*^12^	*U*^13^	*U*^23^
Sn1	0.01931 (11)	0.01664 (11)	0.02035 (12)	0.00686 (9)	−0.00117 (9)	0.00464 (8)
Sn2	0.01779 (11)	0.01694 (11)	0.01666 (11)	0.00366 (8)	−0.00037 (8)	0.00452 (8)
O1	0.0204 (11)	0.0277 (13)	0.0273 (13)	0.0075 (10)	−0.0007 (10)	0.0113 (10)
O2	0.0196 (11)	0.0230 (12)	0.0298 (13)	0.0056 (10)	−0.0013 (10)	0.0074 (10)
O3	0.041 (2)	0.046 (2)	0.0266 (17)	−0.0009 (17)	−0.0004 (15)	0.0138 (15)
O3'	0.0221 (19)	0.044 (3)	0.036 (5)	0.003 (2)	−0.003 (3)	0.026 (3)
O4	0.0210 (11)	0.0227 (12)	0.0245 (12)	0.0057 (10)	0.0002 (10)	0.0094 (10)
O5	0.0206 (11)	0.0269 (13)	0.0316 (14)	0.0073 (10)	0.0030 (10)	0.0118 (11)
O6	0.0325 (14)	0.0387 (16)	0.0363 (15)	0.0159 (12)	−0.0065 (12)	0.0079 (13)
C1	0.0270 (17)	0.0193 (16)	0.0240 (17)	0.0076 (14)	0.0034 (14)	0.0066 (13)
C2	0.0279 (17)	0.0168 (15)	0.0281 (18)	0.0094 (14)	0.0057 (15)	0.0040 (13)
C3	0.0285 (18)	0.0301 (19)	0.0282 (19)	0.0051 (15)	0.0001 (15)	0.0054 (15)
C4	0.040 (2)	0.039 (2)	0.028 (2)	0.0118 (18)	−0.0046 (17)	0.0014 (17)
C5	0.033 (2)	0.039 (2)	0.042 (2)	0.0119 (18)	−0.0027 (18)	−0.0136 (19)
C6	0.027 (2)	0.035 (2)	0.061 (3)	−0.0046 (18)	0.010 (2)	−0.011 (2)
C7	0.035 (2)	0.028 (2)	0.042 (2)	0.0002 (17)	0.0119 (18)	−0.0042 (17)
C8	0.0175 (15)	0.0278 (18)	0.0281 (18)	0.0049 (14)	−0.0012 (14)	0.0100 (14)
C9	0.0166 (15)	0.0241 (17)	0.0289 (18)	0.0038 (13)	−0.0047 (13)	0.0118 (14)
C10	0.044 (2)	0.0256 (19)	0.0279 (19)	0.0109 (17)	0.0025 (17)	0.0039 (15)
C11	0.045 (2)	0.037 (2)	0.0227 (19)	0.0069 (19)	−0.0018 (17)	0.0079 (16)
C12	0.035 (2)	0.034 (2)	0.040 (2)	−0.0020 (18)	−0.0051 (18)	0.0197 (18)
C13	0.036 (2)	0.0196 (18)	0.045 (2)	0.0017 (15)	−0.0015 (18)	0.0097 (17)
C14	0.0256 (17)	0.0260 (18)	0.0277 (18)	0.0050 (14)	0.0021 (14)	0.0046 (14)
C15	0.0278 (17)	0.0216 (18)	0.0275 (19)	0.0091 (14)	−0.0025 (14)	0.0036 (14)
C16	0.0265 (17)	0.0215 (17)	0.0222 (16)	0.0045 (14)	−0.0070 (14)	0.0049 (13)
C17	0.035 (2)	0.0263 (19)	0.029 (2)	0.0102 (16)	0.0051 (15)	0.0043 (15)
C18	0.059 (3)	0.034 (2)	0.040 (2)	0.027 (2)	−0.004 (2)	0.0014 (19)
C19	0.093 (4)	0.020 (2)	0.038 (2)	0.015 (2)	−0.010 (3)	0.0038 (17)
C20	0.075 (3)	0.023 (2)	0.033 (2)	−0.004 (2)	0.005 (2)	0.0032 (17)
C21	0.041 (2)	0.026 (2)	0.032 (2)	−0.0016 (17)	0.0015 (18)	0.0019 (16)
C22	0.0238 (16)	0.0285 (19)	0.0199 (16)	0.0044 (14)	0.0005 (13)	0.0079 (14)
C23	0.0233 (16)	0.0262 (17)	0.0155 (15)	0.0054 (14)	0.0008 (13)	0.0031 (12)
C24	0.0279 (17)	0.0284 (19)	0.0223 (17)	0.0102 (15)	−0.0009 (14)	0.0038 (14)
C25	0.0307 (19)	0.033 (2)	0.0280 (19)	0.0125 (16)	−0.0058 (15)	−0.0006 (15)
C26	0.033 (2)	0.0251 (19)	0.031 (2)	0.0031 (15)	−0.0066 (16)	−0.0036 (15)
C27	0.034 (2)	0.0262 (19)	0.0311 (19)	0.0120 (16)	−0.0054 (16)	0.0006 (15)
C28	0.0276 (18)	0.033 (2)	0.0232 (18)	0.0110 (15)	−0.0019 (14)	0.0015 (15)
C29	0.0225 (16)	0.0270 (18)	0.0181 (16)	0.0055 (14)	−0.0027 (13)	0.0047 (13)
C30	0.0219 (16)	0.0207 (16)	0.0257 (17)	0.0030 (13)	0.0025 (14)	0.0092 (13)
C31	0.0265 (17)	0.0274 (18)	0.0243 (18)	0.0090 (15)	0.0002 (14)	0.0044 (14)
C32	0.047 (2)	0.037 (2)	0.0212 (18)	0.0135 (19)	−0.0035 (17)	0.0076 (16)
C33	0.060 (3)	0.037 (2)	0.0212 (19)	0.015 (2)	0.0035 (19)	0.0045 (16)
C34	0.050 (2)	0.034 (2)	0.035 (2)	0.0215 (19)	0.0165 (19)	0.0049 (17)
C35	0.034 (2)	0.030 (2)	0.034 (2)	0.0153 (16)	0.0045 (16)	0.0132 (16)
C36	0.0299 (18)	0.0206 (17)	0.0274 (18)	0.0061 (14)	−0.0094 (15)	0.0030 (14)
C37	0.0187 (15)	0.0189 (16)	0.0248 (17)	0.0020 (13)	−0.0037 (13)	0.0042 (13)
C38	0.033 (2)	0.031 (2)	0.033 (2)	0.0118 (16)	0.0045 (16)	0.0064 (16)
C39	0.052 (3)	0.033 (2)	0.036 (2)	0.023 (2)	−0.005 (2)	−0.0003 (18)
C40	0.061 (3)	0.0235 (19)	0.036 (2)	0.009 (2)	−0.019 (2)	0.0058 (16)
C41	0.043 (2)	0.038 (2)	0.043 (3)	−0.0029 (19)	0.002 (2)	0.017 (2)
C42	0.0307 (19)	0.030 (2)	0.040 (2)	0.0078 (16)	0.0094 (17)	0.0111 (17)
C43	0.0220 (16)	0.0176 (16)	0.0239 (17)	0.0029 (13)	−0.0036 (13)	0.0058 (13)
C44	0.0213 (16)	0.0223 (17)	0.0323 (19)	0.0028 (14)	−0.0040 (14)	0.0064 (14)
C45	0.0196 (15)	0.0220 (17)	0.0258 (17)	0.0049 (13)	−0.0009 (13)	0.0045 (13)
C46	0.0242 (17)	0.0251 (18)	0.0242 (17)	0.0029 (14)	−0.0038 (14)	0.0070 (14)
C47	0.0221 (19)	0.044 (3)	0.036 (5)	0.003 (2)	−0.003 (3)	0.026 (3)
C48	0.039 (5)	0.074 (6)	0.081 (7)	−0.008 (4)	0.018 (5)	0.002 (6)
C49	0.070 (7)	0.044 (5)	0.022 (5)	−0.010 (5)	0.008 (5)	0.012 (4)
C47'	0.041 (2)	0.046 (2)	0.0266 (17)	−0.0009 (17)	−0.0004 (15)	0.0138 (15)
C48'	0.019 (5)	0.036 (5)	0.010 (5)	0.006 (4)	−0.006 (4)	0.015 (4)
C49'	0.024 (5)	0.027 (5)	0.020 (5)	0.002 (4)	0.007 (4)	0.019 (4)
C50	0.0224 (16)	0.0197 (16)	0.0220 (17)	0.0067 (13)	−0.0009 (13)	0.0021 (13)
C51	0.0218 (16)	0.0200 (16)	0.0257 (17)	0.0086 (13)	−0.0019 (13)	0.0009 (13)
C52	0.0263 (17)	0.0269 (18)	0.0203 (16)	0.0125 (14)	0.0000 (13)	0.0013 (13)
C53	0.0319 (19)	0.032 (2)	0.0254 (18)	0.0171 (16)	0.0036 (15)	0.0071 (15)
C54	0.045 (2)	0.049 (3)	0.031 (2)	0.022 (2)	−0.0010 (18)	0.0159 (19)
C55	0.054 (3)	0.055 (3)	0.030 (2)	0.031 (2)	−0.0021 (19)	0.014 (2)
C56	0.042 (2)	0.055 (3)	0.040 (2)	0.027 (2)	−0.013 (2)	0.006 (2)

### Geometric parameters (Å, °)

**Table:**

Sn1—C15	2.148 (4)	C23—C28	1.397 (5)
Sn1—C8	2.149 (3)	C24—C25	1.381 (5)
Sn1—C1	2.160 (3)	C24—H24	0.9500
Sn1—O1	2.171 (2)	C25—C26	1.391 (6)
Sn1—O5^i^	2.390 (3)	C25—H25	0.9500
Sn2—C36	2.144 (4)	C26—C27	1.390 (5)
Sn2—C29	2.147 (4)	C26—H26	0.9500
Sn2—C22	2.156 (4)	C27—C28	1.384 (5)
Sn2—O4	2.223 (2)	C27—H27	0.9500
Sn2—O2	2.342 (2)	C28—H28	0.9500
O1—C43	1.276 (4)	C29—C30	1.511 (5)
O2—C43	1.264 (4)	C29—H29A	0.9900
O3—C46	1.362 (6)	C29—H29B	0.9900
O3—C49	1.428 (8)	C30—C31	1.388 (5)
O3'—C46	1.365 (8)	C30—C35	1.401 (5)
O3'—C49'	1.422 (8)	C31—C32	1.387 (5)
O4—C50	1.281 (4)	C31—H31	0.9500
O5—C50	1.261 (4)	C32—C33	1.383 (6)
O5—Sn1^ii^	2.390 (2)	C32—H32	0.9500
O6—C56	1.361 (5)	C33—C34	1.374 (6)
O6—C53	1.371 (5)	C33—H33	0.9500
C1—C2	1.491 (5)	C34—C35	1.381 (6)
C1—H1A	0.9900	C34—H34	0.9500
C1—H1B	0.9900	C35—H35	0.9500
C2—C7	1.389 (5)	C36—C37	1.498 (5)
C2—C3	1.399 (5)	C36—H36A	0.9900
C3—C4	1.388 (5)	C36—H36B	0.9900
C3—H3	0.9500	C37—C42	1.386 (5)
C4—C5	1.375 (6)	C37—C38	1.386 (5)
C4—H4	0.9500	C38—C39	1.387 (6)
C5—C6	1.369 (7)	C38—H38	0.9500
C5—H5	0.9500	C39—C40	1.369 (7)
C6—C7	1.389 (6)	C39—H39	0.9500
C6—H6	0.9500	C40—C41	1.370 (7)
C7—H7	0.9500	C40—H40	0.9500
C8—C9	1.508 (5)	C41—C42	1.392 (6)
C8—H8A	0.9900	C41—H41	0.9500
C8—H8B	0.9900	C42—H42	0.9500
C9—C10	1.377 (5)	C43—C44	1.472 (5)
C9—C14	1.384 (5)	C44—C45	1.331 (5)
C10—C11	1.379 (5)	C44—H44	0.9500
C10—H10	0.9500	C45—C46	1.440 (5)
C11—C12	1.390 (6)	C45—H45	0.9500
C11—H11	0.9500	C46—C47	1.358 (7)
C12—C13	1.388 (6)	C46—C47'	1.363 (10)
C12—H12	0.9500	C47—C48	1.392 (8)
C13—C14	1.383 (5)	C47—H47	0.9500
C13—H13	0.9500	C48—C49	1.356 (9)
C14—H14	0.9500	C48—H48	0.9500
C15—C16	1.499 (5)	C49—H49	0.9500
C15—H15A	0.9900	C47'—C48'	1.375 (10)
C15—H15B	0.9900	C47'—H47'	0.9500
C16—C21	1.383 (5)	C48'—C49'	1.362 (9)
C16—C17	1.386 (5)	C48'—H48'	0.9500
C17—C18	1.382 (6)	C49'—H49'	0.9500
C17—H17	0.9500	C50—C51	1.468 (5)
C18—C19	1.371 (7)	C51—C52	1.323 (5)
C18—H18	0.9500	C51—H51	0.9500
C19—C20	1.381 (8)	C52—C53	1.439 (5)
C19—H19	0.9500	C52—H52	0.9500
C20—C21	1.392 (6)	C53—C54	1.359 (6)
C20—H20	0.9500	C54—C55	1.426 (6)
C21—H21	0.9500	C54—H54	0.9500
C22—C23	1.491 (5)	C55—C56	1.324 (7)
C22—H22A	0.9900	C55—H55	0.9500
C22—H22B	0.9900	C56—H56	0.9500
C23—C24	1.392 (5)		
			
C15—Sn1—C8	119.95 (14)	C26—C25—H25	119.8
C15—Sn1—C1	123.54 (13)	C27—C26—C25	119.1 (4)
C8—Sn1—C1	115.20 (14)	C27—C26—H26	120.5
C15—Sn1—O1	93.66 (13)	C25—C26—H26	120.5
C8—Sn1—O1	93.66 (11)	C28—C27—C26	120.1 (4)
C1—Sn1—O1	94.08 (12)	C28—C27—H27	119.9
C15—Sn1—O5^i^	91.05 (12)	C26—C27—H27	119.9
C8—Sn1—O5^i^	78.06 (11)	C27—C28—C23	121.4 (3)
C1—Sn1—O5^i^	88.99 (12)	C27—C28—H28	119.3
O1—Sn1—O5^i^	171.70 (9)	C23—C28—H28	119.3
C36—Sn2—C29	115.80 (14)	C30—C29—Sn2	110.3 (2)
C36—Sn2—C22	129.12 (15)	C30—C29—H29A	109.6
C29—Sn2—C22	115.00 (13)	Sn2—C29—H29A	109.6
C36—Sn2—O4	88.38 (11)	C30—C29—H29B	109.6
C29—Sn2—O4	86.08 (11)	Sn2—C29—H29B	109.6
C22—Sn2—O4	92.31 (11)	H29A—C29—H29B	108.1
C36—Sn2—O2	93.58 (11)	C31—C30—C35	117.9 (3)
C29—Sn2—O2	85.24 (11)	C31—C30—C29	121.2 (3)
C22—Sn2—O2	93.18 (12)	C35—C30—C29	120.8 (3)
O4—Sn2—O2	171.09 (9)	C32—C31—C30	120.7 (3)
C43—O1—Sn1	124.6 (2)	C32—C31—H31	119.6
C43—O2—Sn2	136.2 (2)	C30—C31—H31	119.6
C46—O3—C49	98.6 (6)	C33—C32—C31	120.3 (4)
C46—O3'—C49'	108.3 (7)	C33—C32—H32	119.9
C50—O4—Sn2	123.8 (2)	C31—C32—H32	119.9
C50—O5—Sn1^ii^	139.3 (2)	C34—C33—C32	120.0 (4)
C56—O6—C53	106.0 (3)	C34—C33—H33	120.0
C2—C1—Sn1	111.2 (2)	C32—C33—H33	120.0
C2—C1—H1A	109.4	C33—C34—C35	119.9 (4)
Sn1—C1—H1A	109.4	C33—C34—H34	120.1
C2—C1—H1B	109.4	C35—C34—H34	120.1
Sn1—C1—H1B	109.4	C34—C35—C30	121.3 (4)
H1A—C1—H1B	108.0	C34—C35—H35	119.4
C7—C2—C3	117.6 (4)	C30—C35—H35	119.4
C7—C2—C1	122.6 (3)	C37—C36—Sn2	121.0 (2)
C3—C2—C1	119.8 (3)	C37—C36—H36A	107.1
C4—C3—C2	120.9 (4)	Sn2—C36—H36A	107.1
C4—C3—H3	119.5	C37—C36—H36B	107.1
C2—C3—H3	119.5	Sn2—C36—H36B	107.1
C5—C4—C3	120.6 (4)	H36A—C36—H36B	106.8
C5—C4—H4	119.7	C42—C37—C38	117.8 (3)
C3—C4—H4	119.7	C42—C37—C36	120.8 (3)
C6—C5—C4	119.0 (4)	C38—C37—C36	121.4 (3)
C6—C5—H5	120.5	C37—C38—C39	121.0 (4)
C4—C5—H5	120.5	C37—C38—H38	119.5
C5—C6—C7	121.2 (4)	C39—C38—H38	119.5
C5—C6—H6	119.4	C40—C39—C38	120.5 (4)
C7—C6—H6	119.4	C40—C39—H39	119.7
C6—C7—C2	120.6 (4)	C38—C39—H39	119.7
C6—C7—H7	119.7	C39—C40—C41	119.4 (4)
C2—C7—H7	119.7	C39—C40—H40	120.3
C9—C8—Sn1	120.5 (2)	C41—C40—H40	120.3
C9—C8—H8A	107.2	C40—C41—C42	120.4 (4)
Sn1—C8—H8A	107.2	C40—C41—H41	119.8
C9—C8—H8B	107.2	C42—C41—H41	119.8
Sn1—C8—H8B	107.2	C37—C42—C41	120.8 (4)
H8A—C8—H8B	106.8	C37—C42—H42	119.6
C10—C9—C14	118.4 (3)	C41—C42—H42	119.6
C10—C9—C8	121.3 (3)	O2—C43—O1	122.5 (3)
C14—C9—C8	120.2 (3)	O2—C43—C44	120.3 (3)
C9—C10—C11	121.6 (4)	O1—C43—C44	117.2 (3)
C9—C10—H10	119.2	C45—C44—C43	122.9 (3)
C11—C10—H10	119.2	C45—C44—H44	118.5
C10—C11—C12	119.8 (4)	C43—C44—H44	118.5
C10—C11—H11	120.1	C44—C45—C46	124.2 (3)
C12—C11—H11	120.1	C44—C45—H45	117.9
C13—C12—C11	118.9 (4)	C46—C45—H45	117.9
C13—C12—H12	120.6	C47—C46—O3	120.5 (6)
C11—C12—H12	120.6	C47'—C46—O3'	105.1 (9)
C14—C13—C12	120.4 (4)	C47—C46—C45	116.4 (5)
C14—C13—H13	119.8	O3—C46—C45	123.0 (4)
C12—C13—H13	119.8	C47'—C46—C45	117.2 (8)
C9—C14—C13	120.7 (4)	O3'—C46—C45	137.6 (5)
C9—C14—H14	119.6	C46—C47—C48	99.0 (6)
C13—C14—H14	119.6	C46—C47—H47	130.5
C16—C15—Sn1	111.8 (2)	C48—C47—H47	130.5
C16—C15—H15A	109.2	C49—C48—C47	112.1 (8)
Sn1—C15—H15A	109.2	C49—C48—H48	124.0
C16—C15—H15B	109.2	C47—C48—H48	124.0
Sn1—C15—H15B	109.2	C48—C49—O3	109.8 (7)
H15A—C15—H15B	107.9	C48—C49—H49	125.1
C21—C16—C17	118.4 (4)	O3—C49—H49	125.1
C21—C16—C15	120.5 (4)	C46—C47'—C48'	113.2 (13)
C17—C16—C15	121.1 (3)	C46—C47'—H47'	123.4
C18—C17—C16	120.9 (4)	C48'—C47'—H47'	123.4
C18—C17—H17	119.5	C49'—C48'—C47'	104.7 (12)
C16—C17—H17	119.5	C49'—C48'—H48'	127.7
C19—C18—C17	120.2 (4)	C47'—C48'—H48'	127.7
C19—C18—H18	119.9	C48'—C49'—O3'	108.6 (10)
C17—C18—H18	119.9	C48'—C49'—H49'	125.7
C18—C19—C20	119.9 (4)	O3'—C49'—H49'	125.7
C18—C19—H19	120.1	O5—C50—O4	121.8 (3)
C20—C19—H19	120.1	O5—C50—C51	120.2 (3)
C19—C20—C21	119.8 (4)	O4—C50—C51	118.0 (3)
C19—C20—H20	120.1	C52—C51—C50	122.9 (3)
C21—C20—H20	120.1	C52—C51—H51	118.6
C16—C21—C20	120.7 (4)	C50—C51—H51	118.6
C16—C21—H21	119.6	C51—C52—C53	124.9 (3)
C20—C21—H21	119.6	C51—C52—H52	117.6
C23—C22—Sn2	107.6 (2)	C53—C52—H52	117.6
C23—C22—H22A	110.2	C54—C53—O6	109.8 (3)
Sn2—C22—H22A	110.2	C54—C53—C52	132.9 (4)
C23—C22—H22B	110.2	O6—C53—C52	117.3 (3)
Sn2—C22—H22B	110.2	C53—C54—C55	106.0 (4)
H22A—C22—H22B	108.5	C53—C54—H54	127.0
C24—C23—C28	117.7 (3)	C55—C54—H54	127.0
C24—C23—C22	122.4 (3)	C56—C55—C54	106.7 (4)
C28—C23—C22	119.7 (3)	C56—C55—H55	126.6
C25—C24—C23	121.3 (3)	C54—C55—H55	126.6
C25—C24—H24	119.4	C55—C56—O6	111.4 (4)
C23—C24—H24	119.4	C55—C56—H56	124.3
C24—C25—C26	120.4 (4)	O6—C56—H56	124.3
C24—C25—H25	119.8		
			
C15—Sn1—O1—C43	61.3 (3)	C35—C30—C31—C32	−1.0 (6)
C8—Sn1—O1—C43	−178.3 (3)	C29—C30—C31—C32	175.8 (4)
C1—Sn1—O1—C43	−62.7 (3)	C30—C31—C32—C33	−0.1 (6)
C36—Sn2—O2—C43	96.2 (3)	C31—C32—C33—C34	0.8 (7)
C29—Sn2—O2—C43	−148.2 (3)	C32—C33—C34—C35	−0.3 (7)
C22—Sn2—O2—C43	−33.4 (3)	C33—C34—C35—C30	−0.9 (7)
C36—Sn2—O4—C50	−57.5 (3)	C31—C30—C35—C34	1.5 (6)
C29—Sn2—O4—C50	−173.5 (3)	C29—C30—C35—C34	−175.3 (4)
C22—Sn2—O4—C50	71.6 (3)	C29—Sn2—C36—C37	−115.0 (3)
C15—Sn1—C1—C2	167.6 (2)	C22—Sn2—C36—C37	68.5 (4)
C8—Sn1—C1—C2	0.7 (3)	O4—Sn2—C36—C37	160.1 (3)
O1—Sn1—C1—C2	−95.3 (2)	O2—Sn2—C36—C37	−28.6 (3)
O5^i^—Sn1—C1—C2	77.0 (2)	Sn2—C36—C37—C42	−79.8 (4)
Sn1—C1—C2—C7	−105.6 (4)	Sn2—C36—C37—C38	102.9 (4)
Sn1—C1—C2—C3	73.7 (4)	C42—C37—C38—C39	−1.6 (6)
C7—C2—C3—C4	−1.7 (6)	C36—C37—C38—C39	175.8 (4)
C1—C2—C3—C4	178.9 (3)	C37—C38—C39—C40	−0.1 (7)
C2—C3—C4—C5	0.5 (6)	C38—C39—C40—C41	1.9 (7)
C3—C4—C5—C6	0.8 (7)	C39—C40—C41—C42	−2.0 (7)
C4—C5—C6—C7	−0.9 (7)	C38—C37—C42—C41	1.5 (6)
C5—C6—C7—C2	−0.4 (7)	C36—C37—C42—C41	−175.9 (4)
C3—C2—C7—C6	1.6 (6)	C40—C41—C42—C37	0.3 (7)
C1—C2—C7—C6	−179.0 (4)	Sn2—O2—C43—O1	159.9 (2)
C15—Sn1—C8—C9	76.6 (3)	Sn2—O2—C43—C44	−19.5 (5)
C1—Sn1—C8—C9	−116.0 (3)	Sn1—O1—C43—O2	3.1 (5)
O1—Sn1—C8—C9	−19.7 (3)	Sn1—O1—C43—C44	−177.5 (2)
O5^i^—Sn1—C8—C9	160.9 (3)	O2—C43—C44—C45	172.4 (3)
Sn1—C8—C9—C10	94.1 (4)	O1—C43—C44—C45	−7.1 (5)
Sn1—C8—C9—C14	−89.0 (4)	C43—C44—C45—C46	−175.2 (3)
C14—C9—C10—C11	−2.2 (6)	C49—O3—C46—C47	0.2 (4)
C8—C9—C10—C11	174.8 (4)	C49—O3—C46—C47'	−177 (8)
C9—C10—C11—C12	−0.5 (7)	C49—O3—C46—O3'	−0.4 (7)
C10—C11—C12—C13	2.4 (6)	C49—O3—C46—C45	−178.2 (4)
C11—C12—C13—C14	−1.6 (6)	C49'—O3'—C46—C47	−179 (2)
C10—C9—C14—C13	3.0 (5)	C49'—O3'—C46—O3	−0.5 (8)
C8—C9—C14—C13	−174.0 (3)	C49'—O3'—C46—C47'	−0.2 (4)
C12—C13—C14—C9	−1.1 (6)	C49'—O3'—C46—C45	176.7 (6)
C8—Sn1—C15—C16	−19.5 (3)	C44—C45—C46—C47	6.9 (6)
C1—Sn1—C15—C16	174.2 (2)	C44—C45—C46—O3	−174.7 (5)
O1—Sn1—C15—C16	76.9 (3)	C44—C45—C46—C47'	−174.8 (7)
O5^i^—Sn1—C15—C16	−96.3 (3)	C44—C45—C46—O3'	8.6 (10)
Sn1—C15—C16—C21	−80.7 (4)	O3—C46—C47—C48	−0.2 (2)
Sn1—C15—C16—C17	98.1 (4)	C47'—C46—C47—C48	0.1 (10)
C21—C16—C17—C18	−2.0 (6)	O3'—C46—C47—C48	1.5 (19)
C15—C16—C17—C18	179.1 (4)	C45—C46—C47—C48	178.3 (4)
C16—C17—C18—C19	0.8 (7)	C46—C47—C48—C49	0.0 (2)
C17—C18—C19—C20	0.7 (7)	C47—C48—C49—O3	0.1 (4)
C18—C19—C20—C21	−1.0 (7)	C46—O3—C49—C48	−0.1 (4)
C17—C16—C21—C20	1.7 (6)	C47—C46—C47'—C48'	0.6 (11)
C15—C16—C21—C20	−179.4 (4)	O3—C46—C47'—C48'	3(8)
C19—C20—C21—C16	−0.2 (7)	O3'—C46—C47'—C48'	0.1 (3)
C36—Sn2—C22—C23	177.3 (2)	C45—C46—C47'—C48'	−177.6 (4)
C29—Sn2—C22—C23	0.8 (3)	C46—C47'—C48'—C49'	0.1 (3)
O4—Sn2—C22—C23	87.5 (2)	C47'—C48'—C49'—O3'	−0.2 (4)
O2—Sn2—C22—C23	−85.4 (2)	C46—O3'—C49'—C48'	0.3 (6)
Sn2—C22—C23—C24	96.7 (3)	Sn1^ii^—O5—C50—O4	−160.6 (2)
Sn2—C22—C23—C28	−79.2 (4)	Sn1^ii^—O5—C50—C51	20.7 (5)
C28—C23—C24—C25	−1.0 (5)	Sn2—O4—C50—O5	−16.0 (5)
C22—C23—C24—C25	−176.9 (3)	Sn2—O4—C50—C51	162.7 (2)
C23—C24—C25—C26	0.2 (6)	O5—C50—C51—C52	178.5 (3)
C24—C25—C26—C27	0.0 (6)	O4—C50—C51—C52	−0.2 (5)
C25—C26—C27—C28	0.7 (6)	C50—C51—C52—C53	−176.2 (3)
C26—C27—C28—C23	−1.6 (6)	C56—O6—C53—C54	0.7 (4)
C24—C23—C28—C27	1.7 (5)	C56—O6—C53—C52	179.7 (3)
C22—C23—C28—C27	177.7 (3)	C51—C52—C53—C54	−179.2 (4)
C36—Sn2—C29—C30	−8.7 (3)	C51—C52—C53—O6	2.1 (5)
C22—Sn2—C29—C30	168.4 (2)	O6—C53—C54—C55	−0.3 (5)
O4—Sn2—C29—C30	77.7 (2)	C52—C53—C54—C55	−179.1 (4)
O2—Sn2—C29—C30	−100.3 (2)	C53—C54—C55—C56	−0.2 (5)
Sn2—C29—C30—C31	−95.1 (3)	C54—C55—C56—O6	0.7 (5)
Sn2—C29—C30—C35	81.6 (4)	C53—O6—C56—C55	−0.9 (5)

Symmetry codes: (i) *x*−1, *y*, *z*; (ii) *x*+1, *y*, *z*.
